# Comparison of Dried Blood Spot Sampling Methods for RNA‐Based Biomarker Measurement in Anti‐Doping

**DOI:** 10.1002/dta.70091

**Published:** 2026-05-20

**Authors:** Jacob Bejder, Corentin Schlechten, Claudia Mumenthaler, Olivier Salamin, Tiia Kuuranne, Thomas Christian Bonne, Jonathan Graae, Henrik Sørensen, Nikolai B. Nordsborg, Nicolas Leuenberger

**Affiliations:** ^1^ Department of Nutrition, Exercise and Sports (NEXS) University of Copenhagen Copenhagen Denmark; ^2^ Swiss Laboratory for Doping Analyses University Center of Legal Medicine, Lausanne and Geneva, Lausanne University Hospital and University of Lausanne Lausanne Switzerland; ^3^ Swiss Sport Integrity Bern Switzerland; ^4^ Department of Anesthesia Herlev/Gentofte Hospital Herlev Denmark

**Keywords:** *ALAS2*, anti‐doping, *CA1*, dried blood sampling, erythropoietin, RNA biomarkers, Tasso‐M20

## Abstract

Dried blood spot (DBS) sampling offers logistical advantages, but it remains unexplored whether analytical results are comparable across DBS sampling methods. This study investigated whether venous or capillary blood spotted on cellulose cards or collected with the Tasso‐M20 device provides comparable results for the anti‐doping RNA‐based biomarkers of erythropoiesis, 5‐aminolevulinic acid synthase (*ALAS2*), and carbonic anhydrase 1 (*CA1*). In the population of Swiss athletes (*n* = 12), DBS samples (venous‐cellulose and Tasso‐M20) were collected in routine anti‐doping procedures. In healthy volunteers (*n* = 12), DBS samples (venous‐cellulose, capillary‐cellulose and Tasso‐M20) were collected at baseline, after 1 week and 10 days post a 3‐week recombinant human erythropoietin (rEPO) administration study. *ALAS2* and *CA1* mRNA were quantified using RT‐qPCR, and agreement between matrices was assessed via Passing–Bablok regression. *ALAS2* and *CA1* expression showed strong linear agreement (*r* ≥ 0.96) across matrices. Passing–Bablok regression indicated no constant or proportional bias for *ALAS2* across all comparisons. For *CA1*, no bias existed between venous‐cellulose and Tasso‐M20 in the athlete population, whereas a proportional bias of ~9%–22% was observed when comparing Tasso‐M20 DBS with venous‐ or capillary‐cellulose DBS in the rEPO study. No significant differences in relative RNA expression were observed across matrices at any timepoint in the administration study. *ALAS2* and *CA1* are reliably quantified across venous‐ and capillary‐cellulose‐based DBS and Tasso‐M20 DBS samples. Strong agreement and minimal bias, with the modest *CA1* bias being small relative to expected biological or treatment‐induced variability, support their use for longitudinal monitoring, enabling less invasive, flexible, and decentralized sampling in research and anti‐doping.

## Graphical abstract

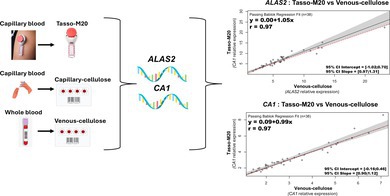

## 1 Introduction

For several years, two erythropoiesis‐related mRNA biomarkers, 5‐aminolevulinic acid synthase (*ALAS2*) and carbonic anhydrase 1 (*CA1*) genes, have been recognized as sensitive markers of blood doping. Both biomarkers can assist to sensitively detect microdose administration of recombinant erythropoietin (rEPO) [1] and are downregulated after a 450‐mL blood donation or a 280‐mL transfusion of packed red blood cells [2, 3]. Furthermore, *ALAS2* and *CA1* appear to be less influenced by confounding factors such as altitude exposure and iron injection than currently established biomarkers of blood doping in athlete biological passport, e.g., the reticulocyte percentage (RET%) [1, 4]. Indeed, they represent complementary biomarkers that may enhance the athlete biological passports capability to capture subtle or transient physiological effects associated with blood manipulation, thereby strengthening the overall framework for longitudinal monitoring of athletes.

One major advantage of the mRNA biomarkers is the option of reliably quantifying their level in dried blood spots (DBS) [5]. DBS has numerous advantages compared to traditional venous blood sampling. For instance, the collection is less invasive, and the sample has higher mRNA stability at room temperature. As a result, DBS samples—whether obtained from capillary blood (finger‐prick) or from venous blood spotted onto DBS cards—can be shipped without strict time or temperature constraints, substantially reducing logistical complexity and shipping costs [5, 6, 7]. Moreover, DBS is preferred over venous blood collection by athletes and doping control officers [8]. DBS has also been successfully applied for routine anti‐doping analyses [9, 10, 11] and is already regulated in a first technical document published by WADA in 2021 for qualitative analyses [12].

The incorporation of *ALAS2* and *CA*1 into standard anti‐doping testing protocols and the athlete biological passport workflow using routine EDTA blood samples to generate venous DBS samples has already been demonstrated without significant logistical or analytical constraints [13]. Nevertheless, DBS samples can also be generated directly using various collection approaches, which may differ with respect to blood volume control, user dependency, or analytical variability. One approach is the conventional finger‐prick sampling, in which a few drops of capillary blood are deposited onto adapted filter paper. More recently, automated capillary blood collection devices such as the Tasso‐M20 have emerged and are increasingly integrated into anti‐doping testing strategies. The Tasso‐M20 is a small, self‐contained device that is typically placed on the upper arm, where it uses a gentle vacuum‐based mechanism to collect a metered volume of capillary blood (17.5 μL) without the need for trained phlebotomists [8]. The Tasso‐M20 system offers several advantages compared with conventional finger‐prick sampling. First, it standardizes the volume of blood collected, reducing variability linked to manual spotting. Second, athletes can perform the collection themselves, even remotely, which could enable decentralized testing schemes and increase the frequency and flexibility of sample collection [14]. The process is largely pain‐free, minimally invasive, and more acceptable to athletes than traditional venipuncture, thereby improving compliance [8]. Studies have demonstrated that the Tasso‐M20 produces DBS samples of sufficient quality for a range of analytical applications, including anti‐doping molecular assays [9, 10, 15, 16].

Due to the promising outlook for *ALAS2* and *CA1* genes as transcriptomic biomarkers of blood doping, it is important to determine whether analytical results are comparable across methods of DBS collection. In this study, we therefore compared the expression of *ALAS2* and *CA1* in three different dried blood matrices: the conventional capillary finger‐prick with manual spotting on cellulose‐based filter paper, capillary blood collected using the Tasso M20 device, and finally venous blood manually spotted on cellulose‐based filter paper. We hypothesized that the relative expression levels of *ALAS2* and *CA1* would be comparable across biological matrices.

## 2 Materials and Methods

The present study utilized samples collected in two different studies. The first study was a human clinical trial with rEPO administration conducted at the Department of Nutrition, Exercise, and Sports, University of Copenhagen, whereas the second study included samples from elite athletes that had been collected by Swiss Sport Integrity (SSI).

### 2.1 Athletes' Population

**FIGURE 1:**
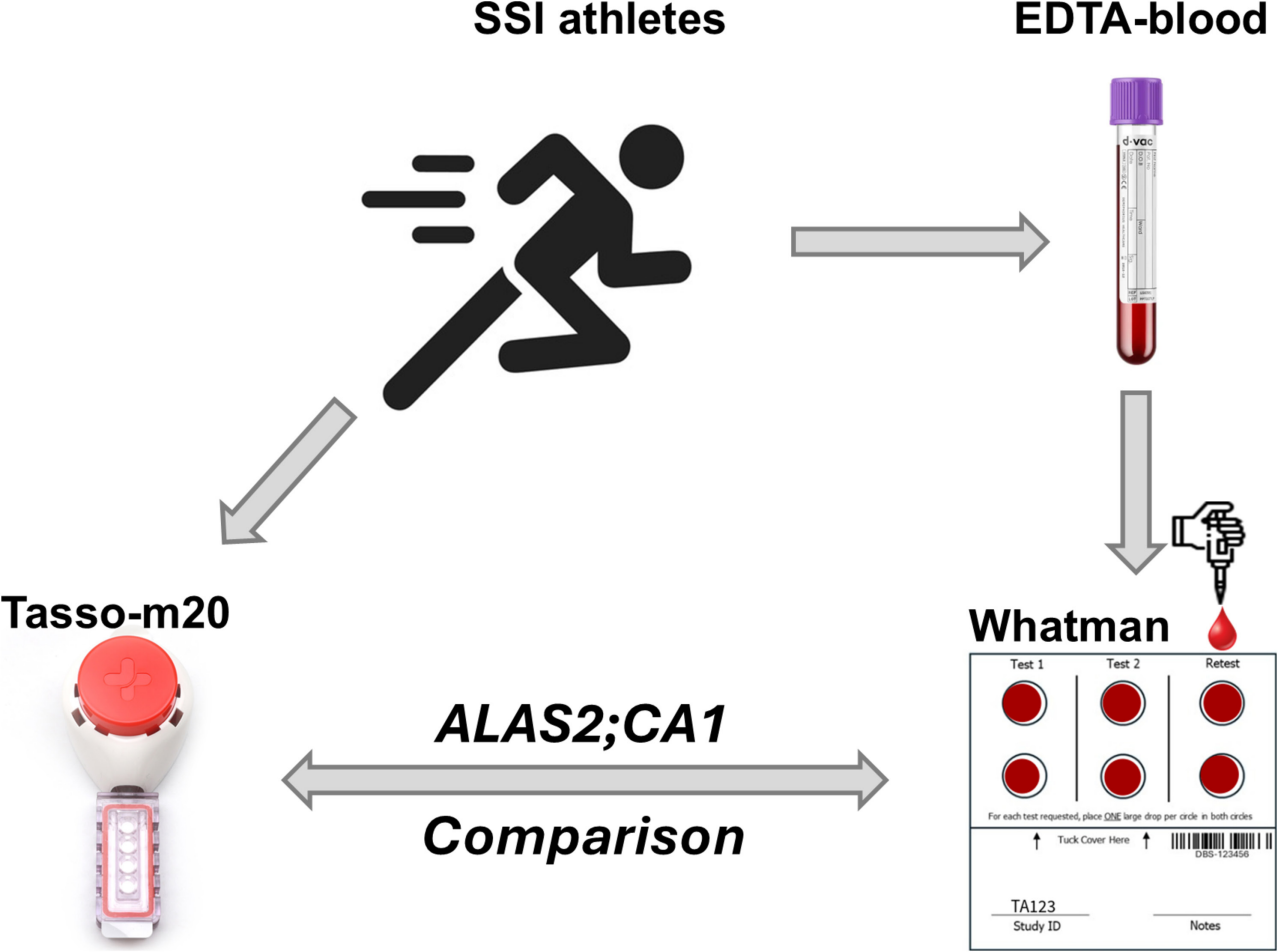
Overview of the study comparing dried blood microsampling devices for RNA‐based biomarker analysis in elite athletes. Schematic of the workflow comparing Whatman cards spotted with venous EDTA blood and the Tasso‐M20 device. Dried blood samples were analyzed by qPCR for mRNA biomarkers, and the outputs were compared to evaluate the interchangeability of dried blood microsampling for anti‐doping applications.

SSI selected six male and six female professional athletes mainly involved in endurance disciplines who underwent venous blood collection in EDTA tubes as well as DBS sample using the Tasso‐M20 microneedle‐based device as part of the regular anti‐doping procedures. Once the samples were received at the laboratory, 20 μL of each EDTA sample was spotted per spot on Whatman 903 saver cards to generate five spots per sample. After drying for 1 h, the venous‐cellulose DBS samples were stored at 4°C with desiccant bags until analysis. The Tasso‐M20 samples were stored at −20°C upon receipt until analysis (Figure 1). For each athlete, three to four blood samples were collected over 6 months. The study included athletes of both sexes and from various sports disciplines (Table 1).

**TABLE 1:** Athletes, sex, sport disciplines, and timepoints investigated in the study.

Sports	Sex	Timepoints
Cycling	2 M/2 W	3–4
Aquatics/swimming middle distance	2 M	3–4
Athletics/long distance (3000 m or greater)	2 W	3–4
Biathlon	2 W	3–4
Skiing	2 M	3

Abbreviations: M, males; W, women.

### 2.2 Recombinant EPO (rEPO) Administration Study

Twelve nonsmoking and recreationally active males, aged between 18 and 40 years, participated in the study. The participants were sea‐level residents, had not donated blood for at least 3 months before the study, and were asked to maintain their normal physical activity during the study period to reduce confounding. The study was approved by the Copenhagen ethics committee (H‐20064997) and conducted in accordance with the Declaration of Helsinki. Participants provided written informed consent after receiving oral and written information on potential risks.

**FIGURE 2:**
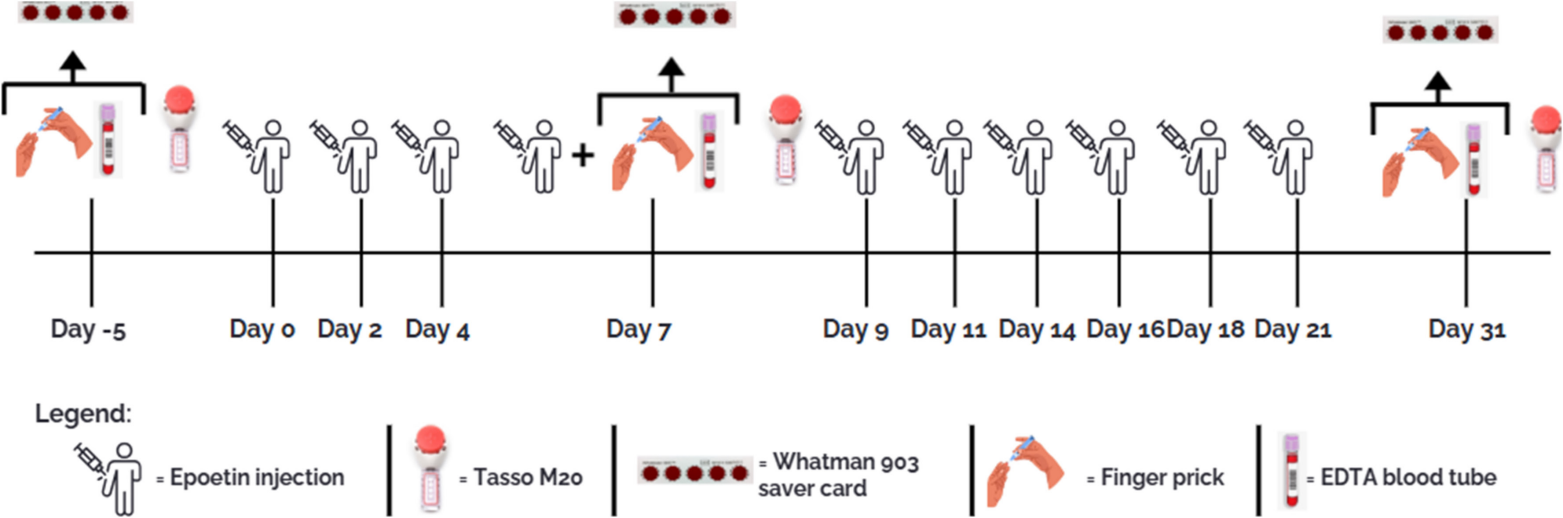
Experimental design of rEPO administration study. Participants received subcutaneous epoetin injections on Days 0, 2, 4, 7, 9, 11, 14, 16, and 18. Capillary and venous blood samples were collected at Days −5, 7, and 28 (i.e., 10 days after last injection) using three sampling modalities: Tasso M20 devices, finger‐prick collection onto *Whatman* 903 protein saver cards (dried blood spots), and EDTA venous blood tubes. Venous EDTA blood was also spotted onto *Whatman* 903 cards for parallel dried blood spot analysis. The upper panels illustrate the sampling type performed on each study day.

Participants completed a 7‐week study period, which included a 2‐week baseline, a 3‐week intervention, and a 2‐week follow‐up. During the 3‐week intervention, participants received subcutaneous injections of 50‐IU·kg^−1^ epoetin beta (NeoRecormon, Roche, Germany) every Monday, Wednesday, and Friday. DBS and EDTA whole blood samples were collected 5 days before administration initiation (baseline), after 7 days (T7) of treatment, and 10 days (T10) after the end of the treatment (Figure 2).

DBS samples were collected by three different methods (Figure 2):

- Spotting of 20‐μL venous blood collected in EDTA tubes onto Whatman 903 saver card with micropipette.
- Spotting of capillary 20‐μL blood obtained from finger‐prick onto Whatman 903 saver card with micropipette.
- Application of microneedle‐based device (Tasso‐M20) on upper arm (17.5 μL).

The collection and analysis of these samples followed World Anti‐Doping Agency (WADA) guidelines. The DBS samples were collected in November 2022 and stored at room temperature in ziplock bags with desiccant for 2.5 years prior to shipment to the Swiss Laboratory for Doping Analyses. During transport, the samples were maintained at ambient temperature and subsequently stored at 4°C upon arrival until analysis.

### 2.3 Erythropoiesis‐Related mRNA Extraction From DBS Samples and Analysis

Extraction of erythropoiesis‐related mRNA from DBS samples and subsequent reverse transcriptase (RT)‐quantitative polymerase chain reaction (qPCR) analysis were performed as described previously [1, 3, 13, 17]. DBS were equilibrated at ambient temperature (20°C–25°C) for 30 min prior to initiating the extraction procedure. Briefly, DBS were excised and lysed in phenol/guanidine (Qiagen), followed by sonication and chloroform extraction. The aqueous phase was processed using the Maxwell RSC system with the miRNA Plasma and Serum Kit (Promega) for automated RNA purification.

cDNA was synthesized from 50‐ to 100‐ng RNA using the Transcriptor First Strand kit (Roche) with random hexamers; NRT controls were included.

RT‐qPCR was performed on a LightCycler 480 (Roche) using SYBR Green chemistry (Qiagen). Three housekeeping genes (*GAPDH*, *RGCC L*, and *RGCC C*) were used to normalize the results.

### 2.4 Statistics

Before performing statistical analyses, normality of the data was assessed using the Shapiro–Wilk test. To assess differences in RNA relative expression between DBS matrices, the Friedman test, serving as a nonparametric alternative to repeated‐measures ANOVA, was applied separately at each of the timepoints as the data were not normally distributed. This test was used for both rEPO administration study and samples for athlete population. Moreover, Passing–Bablok regression, a robust nonparametric method, was also used to evaluate visually and statistically agreement between matrices. Passing–Bablok regression was performed to calculate the slope, intercept, their 95% confidence intervals (CIs), and the correlation coefficient. Absence of constant or proportional bias was assumed when the 95% CI of the intercept included zero, and that of the slope included one. Statistical significance was set at *p* < 0.05. *ALAS2* was evaluated as the mean of *ALAS2LC* and *ALAS2L*. Statistical analyses were performed using RStudio software.

## 3 Results

### 3.1 Comparison of Venous‐Cellulose‐Based DBS and Tasso‐M20 Samples for *ALAS2* and *CA1* Analysis in the Athlete's Population

**FIGURE 3:**
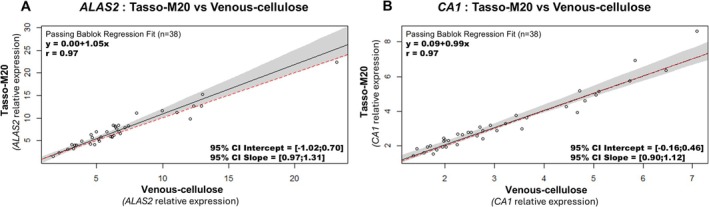
Comparison of dried blood matrices in athletes' population. Passing–Bablok regression comparing Tasso‐M20 and venous‐cellulose blood on Whatman saver card for *ALAS2* (A) and *CA1* (B). Black line indicates regression line, red dashed line indicates identity line, and the confidence bands for regression are shown in gray. The correlation coefficient is indicated by *r*, and 95% CI corresponds to the 95% confidence interval.

Passing–Bablok regression showed strong agreement (*r* = 0.97) between venous‐cellulose and Tasso‐M20 samples for the *ALAS2* and *CA1* biomarkers (Figure 3A,B). The slopes close to unity, and the narrow CIs support the interchangeability of venous‐cellulose and Tasso‐M20 sampling for reliable quantification of *ALAS2* and *CA1* with indications of insignificant proportional bias of only 5% and 1%, respectively (Figure 3A,B). This was further supported by the absence of significant differences in the mean relative expression level between the sampling methods.

### 3.2 Comparison of Venous‐ and Capillary‐Cellulose‐Based DBS and Tasso‐M20 Samples for *ALAS2* and *CA1* Analysis in a rEPO Administration Cohort

**FIGURE 4:**
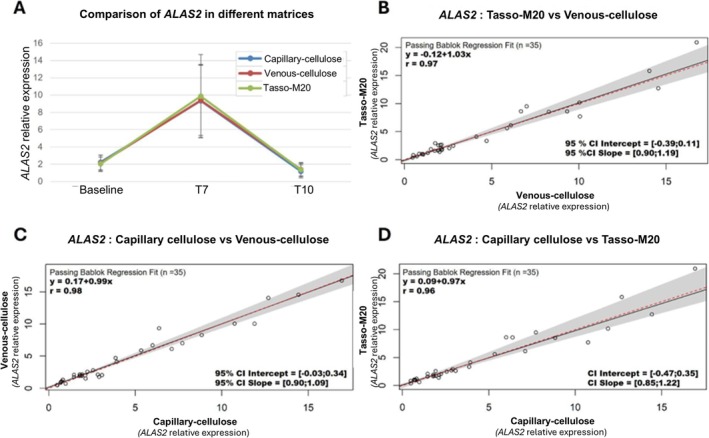
Comparison of dried blood matrices following epoetin beta injection for *ALAS2.* (A) Relative *ALAS2* expression measured in Whatman 903 saver cards using capillary‐cellulose blood (blue line), in Whatman 903 saver cards using venous‐cellulose blood (red line), and in the Tasso‐M20 device (green line). Baseline corresponds to the sample collected 5 days prior to treatment initiation. T7 refers to the sample collected after 1 week of treatment, and T10 corresponds to the sample collected 10 days after treatment cessation. Error bars represent standard deviation. (B–D) Passing–Bablok regression analyses comparing the different matrices. Black line indicates regression line, red dashed line indicates identity line, and the confidence bands for regression are shown in gray. The correlation coefficient is indicated by *r*, and 95% CI corresponds to the 95% confidence interval.

For *ALAS2* (Figure 4A), there were no significant differences in the mean relative expression level between matrices at baseline (venous‐cellulose: 2.0 ± 0.9, capillary‐cellulose: 2.2 ± 0.8, Tasso‐M20: 2.0 ± 0.8) or with accelerated erythropoiesis at T7 (venous‐cellulose: 9.4 ± 4.1, capillary‐cellulose: 9.3 ± 4.0, Tasso‐M20: 9.9 ± 4.7) or decelerated erythropoiesis at T10 (venous‐cellulose: 1.4 ± 0.8, capillary‐cellulose: 1.2 ± 0.8, Tasso‐M20: 1.4 ± 0.7). Moreover, the slopes, CIs, and strong correlations (*r* ≥ 0.96 for all comparisons) observed with the Passing–Bablok regression (Figure 4B–D) indicate insignificant bias across matrices of only 3% between venous‐cellulose and Tasso‐M20, 1% between capillary‐cellulose and venous‐cellulose, and 3% between capillary‐cellulose and Tasso‐M20.

**FIGURE 5:**
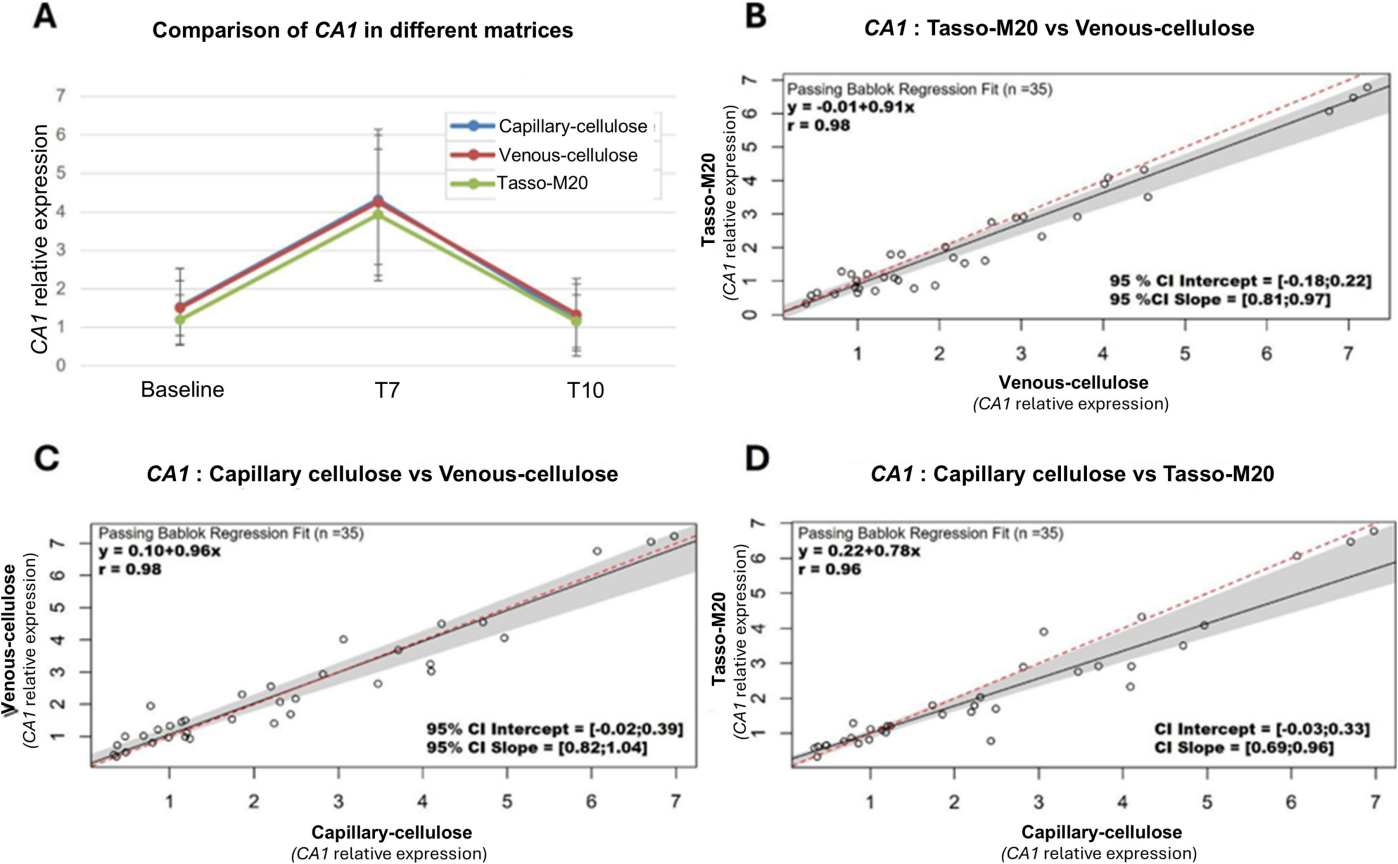
Comparison of dried blood matrices following epoetin beta injection for *CA1.* (A) Relative *CA1* expression measured in Whatman cards using capillary blood (blue line), in Whatman cards using venous blood (red line), and in Tasso‐M20 device (green line). Baseline corresponds to the sample collected 5 days prior to treatment initiation. T7 refers to the sample collected after 1 week of treatment, and T10 corresponds to the sample collected 10 days after treatment cessation. Error bars represent standard deviation. (B–D) Passing–Bablok regression analyses comparing the different matrices. Black line indicates regression line, red dashed line indicates identity line, and the confidence bands for regression are shown in gray. The correlation coefficient is indicated by *r*, and 95% CI corresponds to the 95% confidence interval.

Similarly, for *CA1* (Figure 5A), there were no significant differences in the mean relative expression level between matrices at baseline (venous‐cellulose: 1.5 ± 0.7, capillary‐cellulose: 1.5 ± 1.0, Tasso‐M20: 1.2 ± 0.7) or with accelerated erythropoiesis at T7 (venous‐cellulose: 4.3 ± 1.9, capillary‐cellulose: 4.3 ± 1.7, Tasso‐M20: 3.9 ± 1.7) or decelerated erythropoiesis at T10 (venous‐cellulose: 1.3 ± 0.9, capillary‐cellulose: 1.2 ± 0.9, Tasso‐M20: 1.2 ± 0.7). The Passing–Bablok regression for *CA1* (Figure 5B–D), however, revealed a global bias of 9% between venous‐cellulose and Tasso‐M20 and 22% between capillary‐cellulose and Tasso‐M20. In contrast, the capillary‐cellulose versus venous‐cellulose DBS comparison showed no proportional bias (4%). Moreover, strong correlations were observed across all matrices (*r* ≥ 0.96).

## 4 Discussion

The use of DBS offers a practical, efficient, and minimally invasive method compared to conventional venipuncture. In the context of athlete monitoring, reduced invasiveness is particularly advantageous, as it may facilitate more frequent or decentralized sampling. In addition to the ease of collection, DBS provides logistical benefits, as transport and storage are considerably simpler than for whole blood samples [18]. Previous studies have demonstrated the suitability of DBS for the direct detection of blood doping [19] and indirect detection strategies via, e.g., quantification of protein [20] or transcriptomic [21, 22] biomarkers. However, quantitative results may vary depending on the DBS sampling procedure. The present study extends previous work by systematically evaluating the interchangeability of DBS samples collected by spotting venous or capillary blood spotted on cellulose card or obtained using the Tasso‐M20 device for quantification of the transcriptomic biomarkers *ALAS2* and *CA1*. The major finding is that the evaluated sampling approaches in general yielded comparable results across matrices, both under physiological conditions in a real‐world athlete population (venous‐cellulose vs. Tasso‐M20) and during pharmacologically stimulated erythropoiesis in a controlled human clinical trial (all matrices). The matrices provided strong linear associations (*r* ≥ 0.96) and minimal proportional bias (1%–22%), supporting the analytical comparability of the matrices for RNA‐based biomarker assessment. For *CA1*, a modest proportional bias (9%–22%) was observed by the Tasso‐M20 and the two cellulose‐based DBS matrices. However, this is small relative to the magnitude of biological and treatment‐induced variability, indicating the bias is unlikely to affect interpretation.

### 4.1 Bias Between Sampling Procedures

While the slopes and CIs for *ALAS2* indicated the absence of constant and proportional bias across all comparisons, a minor proportional bias was observed for *CA1*. Specifically, when the Tasso‐M20 samples were compared to venous‐ or capillary‐cellulose samples, the Passing–Bablok regressions indicated that Tasso‐M20 samples yielded slightly lower *CA1* values, corresponding to a proportional bias of 9% and 22% (Figure 5B,D). However, the magnitude of this bias should be interpreted in the context of biological variability and treatment‐induced changes. In the present study, 3 weeks of rEPO treatment (50‐IU/kg bw, three times per week) induced an ~250%–300% increase in *CA1*. Even 3 weeks of microdose administration (20‐IU/kg bw, every second day) induced an ~200% increase in *CA1* [1]. In addition, exposure to moderate hypoxia increases *CA1* by ~40%–50% [1]. Similarly, longitudinal studies indicate that *CA1* may vary ~30% within weeks and ~40% over several months in authentic athlete populations [4, 13]. Consistent with this, estimates of intraindividual variability for *CA1* range between ~8% and 20% [3]. Taken together, these findings indicate that the magnitude of the proportional bias is considerably less than the expected biological or treatment‐induced variability for *CA1*.

In summary, *ALAS2* was interchangeable across all matrices as was *CA1* for venous and capillary blood spotted on cellulose cards. However, for *CA1* analysis, the Tasso‐M20 device showed a minor proportional difference compared with both capillary and venous blood on cellulose cards. This suggests that the observed difference is primarily related to the collection device rather than the sampling site. From a practical perspective, the proportional bias of 9% and 22% appears small relative to the biological (~30%–50%) and treatment‐induced (~100%–300%) variability and is therefore unlikely to compromise the utility of RNA‐based biomarkers for monitoring erythropoietic stimulation in athlete populations.

### 4.2 Two Cohort Evaluations

By including two complementary cohorts, we were able to evaluate the comparability of DBS sampling approaches across a broad range of physiological conditions. In authentic athlete samples, the biomarkers exhibited natural variability, whereas in the rEPO cohort, pharmacologically induced erythropoietic stimulation produced large and rapid changes. The strong agreement across matrices in both settings indicates that the evaluated sampling approaches are reliable not only under baseline conditions but also when the biomarkers undergo substantial biological changes. This supports the robustness and generalizability of the DBS methods for RNA‐based biomarker assessment in both research and longitudinal monitoring contexts.

## 5 Conclusion

This study demonstrates that RNA‐based biomarkers of erythropoiesis, *ALAS2* and *CA1*, can be reliably quantified across commonly used DBS sampling matrices, including venous and capillary blood spotted on cellulose cards and the Tasso‐M20 device. Strong agreement between matrices and minimal bias relative to biological and treatment‐induced variability support the analytical equivalence of these approaches for longitudinal monitoring. Importantly, the feasibility and flexibility of DBS sampling enable less invasive and potentially more frequent testing, as well as decentralized sample collection outside laboratory or competition settings, without compromising analytical reliability. Together, these findings support the practical implementation of DBS sampling for RNA‐based biomarker assessment in both research and anti‐doping contexts and the potential of *ALAS2* and *CA1* as complementary biomarkers for monitoring erythropoietic activity.

## Funding

We are grateful to Swiss Sport Integrity for financial support and logistical organization.

## Conflicts of Interest

The authors declare no conflicts of interest.

## Data Availability

Data are available on request from the authors.
